# Hand–arm vibration: Swedish carpenters’ perceptions of health and safety management

**DOI:** 10.1093/occmed/kqad013

**Published:** 2023-02-06

**Authors:** K Fisk, C Nordander, Å Ek

**Affiliations:** Ergonomics and Aerosol Technology, Department of Design Sciences, Faculty of Engineering LTH, Lund University, SE-221 00 Lund, Sweden; Occupational and Environmental Medicine, Department of Laboratory Medicine, Faculty of Medicine, Lund University, SE-223 63 Lund, Sweden; Occupational and Environmental Medicine, Department of Laboratory Medicine, Faculty of Medicine, Lund University, SE-223 63 Lund, Sweden; Ergonomics and Aerosol Technology, Department of Design Sciences, Faculty of Engineering LTH, Lund University, SE-221 00 Lund, Sweden

## Abstract

**Background:**

Workers in the construction industry are highly exposed to vibration from handheld equipment, which can have negative effects on the nerves and blood vessels in the hands. Employers in this industry often fail to comply with legislation regarding vibration exposure.

**Aims:**

To assess carpenters’ perceptions of proactive health and safety (H&S) management regarding hand–arm vibration exposure at construction sites in Sweden.

**Methods:**

The carpenters answered a questionnaire on their perception of the implementation of H&S management, on symptoms indicating injury and on the use of vibrating equipment.

**Results:**

One hundred and ninety-four carpenters from 4 construction companies and 18 construction sites completed the questionnaire. Attitudes to H&S management were generally positive. However, 36% of the carpenters reported that the H&S regulations and routines did not function in practice, and 40% claimed they did not receive necessary information on the exposure and effects of vibration. Most respondents (74%) perceived a high risk of injury in general in their work. Younger carpenters, carpenters employed at smaller companies and carpenters with symptoms indicating injury or with higher vibration exposure reported more negative perceptions.

**Conclusions:**

In general, the carpenters were positive about management’s commitment to H&S management. However, the results indicate deficiencies in the way in which this commitment is applied in practice at the workplace. This highlights the importance of raising awareness concerning vibration exposure and possible injuries, and strengthening proactive H&S programmes, especially in smaller companies.

## Teaser

This study examines carpenters’ perceptions of health and safety (H&S) management, especially regarding hand–arm vibration. Carpenters had, in general, a positive perception of management’s commitment to H&S management. However, deficiencies in the practical implementation of H&S measures were reported. Younger carpenters, carpenters working at smaller companies and carpenters with symptoms in their hands or with higher exposure were more negative to the H&S management.

**Key learning points**

**What is already known about this subject:**

- Carpenters are exposed to high levels of hand–arm vibration, which may lead to the development of neurological, vascular and musculoskeletal disorders.
- Employers in the construction sector often fail to comply with vibration regulations, which affects proactive health and safety management.

**What this study adds:**

- In general, carpenters were positive concerning the management’s commitment to health and safety.
- This commitment is not reflected in the way health and safety measures are applied in practice onsite.
- Younger carpenters, carpenters working at smaller companies and carpenters with symptoms in their hands or with higher exposure had a more negative perception of health and safety management.

**What impact this may have on practice or policy:**

- This study, on the perceptions of carpenters concerning health and safety management regarding hand–arm vibration, indicates a lack of health and safety training for workers in the construction industry. Sufficient training is important to raise awareness about vibration exposure and vibration injuries in construction workers.
- There is scope for the improvement of health and safety measures for workers in the construction industry.

## Introduction

A larger proportion of workers in the construction industry are exposed to hand–arm vibration from vibrating equipment than in other industries [1]. Almost half, 45%, use vibrating equipment at least a quarter of their workday [2]. Especially carpenters are exposed to higher levels of hand–arm vibration than other professional groups in industry in general [3].

Exposure to hand–arm vibrations may lead to neurological, vascular and musculoskeletal disorders. This often leads to impaired hand function, dexterity, sensation and strength [4]. This may affect the person’s ability to carry out their work, and ultimately have other life-changing effects, such as the need to leave the profession or poorer quality of life [5].

The European Union Vibration Directive 2002/44/EC regulates the obligations and rights of employers and employees regarding exposure to vibration [6]. The responsibilities of the employer defined in this directive are, among others, to perform risk assessments of vibration exposure and to ensure that workers exposed to vibration receive adequate information on vibration exposure and associated risks [6]. If there is a risk of injury, workers must be offered regular health surveillance. It has been found that some employers in the construction industry fail to comply with these regulations. In inspections targeting vibration, the Swedish Work Environment Authority found deficiencies in compliance with requirements for risk assessment and regular health surveillance [7]. Similar deficiencies have also been noted by occupational health services and companies [8], as well as occupational and environmental medicine clinics in Sweden [9].

Poor company compliance with legislation regarding vibration affects proactive health and safety (H&S) management at construction sites and can also be regarded as an indication of the company’s safety culture. An organization with a good safety culture is often characterized by shared responsibility for the H&S of its members, and the fact that safety is highly prioritized by both management and employees [10,11]. Management may have a significant influence on proactive H&S management, and its involvement is one of the most effective factors facilitating safety [12]. This underlines the importance of commitment and motivation among management to ensure safe and healthy workplaces [13]. Management, foremen and supervisors must regularly talk to employees about working safely [14] and ensure that workers are consulted and involved in H&S management [15,16]. Employers should ensure that workers receive adequate information and training concerning the risks of exposure to vibration. For example, it has been found that groups of construction workers who receive more safety training have a better perception of the likelihood of risks occurring at work and their consequences [14].

Effective vibration injury prevention and robust risk management are hampered by the lack of precise definitions and classification of injuries and diseases [17] and by difficulties in assessing exposure to all vibration that affect humans [18]. Symptoms of vibration injury can appear rapidly, although they usually develop insidiously over a long period. It has been found that neurosensory injury occurs within a shorter time than vascular affection, given equal exposures [4]. This emphasizes the importance of implementing long-term proactive H&S programmes in the construction sector.

In this research project, the occurrence of hand–arm vibration syndrome among Swedish carpenters was investigated. The project also aimed to obtain knowledge on carpenters’ perceptions of proactive H&S management within construction companies and onsite. The aims of this study were: (i) to investigate carpenters’ perceptions of proactive H&S management; (ii) to investigate whether these perceptions are influenced by the individual’s age, the size of the company, amount of daily hand–arm vibration exposure or the presence or absence of symptoms in their hands; and (iii) to investigate whether certain subgroups feel that they can influence their working conditions.

## Methods

Carpenters’ perceptions of H&S management were studied at four different construction companies in southern Sweden: two large and two medium-sized and included a total of 18 construction sites. All the carpenters working at a particular site were invited to participate in the study during the first visit to each site. They were informed that participation was voluntary, and that the results would be anonymous. The study was approved by the Regional Ethics Committee in Lund (No. 2015/875). Written informed consent for participation and the publication of the results were obtained from the carpenters who had agreed to participate at the second visit. They were asked to respond to a questionnaire presented on a tablet computer. The questionnaire contained nine items about symptoms in their hands that indicated nerve affection (e.g. numbness or tingling, reduced sensitivity or dexterity) or vascular injuries (i.e. white fingers when cold or damp). The items about symptoms were obtained from a protocol recommended for epidemiological studies concerning vibration exposure, which has been published as a result of the European project Risk of Occupational Vibration Exposure (VIBRISK) [19]. It also included a varying number of items (3–110) on the use of handheld vibrating equipment, i.e. work tasks, type of equipment used and for how long each equipment was used per day. These items were developed based on clinical experience of assessing carpenters and their exposure. The answers to these items were used to quantify the average daily vibration exposure. The questionnaire also contained 12 items focusing on the carpenters’ perceptions of various aspects of proactive H&S management. The items were extracted from a questionnaire used to assess perceptions of and attitudes to H&S management in industrial sectors, aimed at supporting continuous improvement in companies [20]. This questionnaire was based on aspects whose contexts were selected from previous studies in the research field. Aspects included were working situation, communication, learning, reporting, justness, flexibility, attitudes to safety, safety-related behaviours and risk perception. In the previous study, relevant items were developed to represent each of the aspects through literature reviews, field studies in industrial settings and evaluation of their relevance by leaders in central roles. Pilot studies in industrial settings have previously been performed to test the questionnaire in real situations, and reliability analyses have been conducted. In the current study, the areas included in the questionnaire were management’s commitment to safety, employees’ involvement in H&S measures, H&S information and communication, improvement efforts and risk perception. The items were answered using 5-point scales (‘Never’, ‘Seldom’, ‘Sometimes’, ‘Often’, ‘Always’ or ‘Not at all’, ‘Barely’, ‘A little’, ‘Much’, ‘Very much’). The questionnaire in the current study was tested on two carpenters to ensure that the responders could complete the questionnaire. All carpenters also underwent a medical examination onsite to identify any manifestations of vibration injury [21]. However, the purpose of this paper is to present the findings from the questionnaire study.

Statistical calculations were performed using IBM SPSS Statistics 24. Differences between different subgroups were tested with the Mann–Whitney *U*-test. The significance level was defined as *P* ≤ 0.05, two-tailed. As there is a strong correlation between age and seniority, we chose to use age as a measure of the carpenters’ experience.

## Results

All carpenters at the 18 construction sites visited chose to participate, resulting in a total of 194 participants (193 men and 1 woman; Table 1). This is in line with the overall gender composition in the Swedish construction industry. The daily vibration exposure was calculated based on the results from the questionnaire on the carpenter’s reported estimated daily usage of handheld vibrating equipment. The calculated daily vibration exposure exceeded the limit of A(8) ≥ 5 m/s^2^ for 94 carpenters (48%). Just over half (115, 59%) reported at least one neurosensory symptom, answered ‘yes’ to the question on whether they had white fingers, or both.

**Table 1.** Distribution of gender, age, company size, estimated vibration exposure and symptoms in hands

	Carpenters (*n* = 194)
Women [*n* (%)]	1 (0.5)
Age [years; mean (range)]	40 (17–65)
Employed at a large company [*n* (%)]	90 (46)
Estimated daily exposure, A(8) [m/s^2^; mean (range)]	5.1^a^ (0.6–11)
Symptoms in hands [*n* (%)]	115 (59)

^a^To be compared to a limit value of 5 m/s^2^ and an action value of 2.5 m/s^2^ [6].

The average scores from the 12 questions on proactive H&S management showed that the carpenters generally had a positive view of the company’s H&S management. More than 50% of the carpenters gave a positive response (score of 4 or 5) to 9 of the 12 items. Eight out of 10 carpenters felt that management actively encouraged safe work (79%), and that the foremen considered that safety was part of daily work (81%). However, negative responses were given to three questions by one-third or more of the carpenters (score of 1 or 2): 36% reported that regulations and routines for preventing injuries did not function in practice; 40% responded that they never or seldom received the information they needed regarding vibration exposure to be able to carry out their job in a safe manner; and most respondents (74%) perceived there was a high risk of injury in general in their work.

Many statistically significant differences were found between younger (<40 years) and older carpenters, as can be seen from Table 2. In all instances, older carpenters had more positive perceptions of H&S management than younger ones. The differences in perceptions remained after stratification of the material by company size, estimated exposure and experience of hand symptoms. Carpenters employed by larger companies generally had somewhat more positive perceptions than those employed by medium-sized companies. This pattern remained after stratification by age and exposure, although fewer differences were statistically significant. When stratifying the material according to symptoms/no symptoms, differences in responses depending on the size of the company could mainly be seen in those who reported experiencing symptoms. Almost half of the carpenters (48%) had an estimated daily exposure at or above the limit of 5 m/s^2^. Carpenters with higher exposure generally expressed more negative perceptions of the items than those with a lower estimated daily exposure (8 of 12 instances statistically significant; Table 2). This pattern remained after stratification by age, company size and symptoms/no symptoms, although fewer differences were statistically significant. Just over half of the carpenters (59%) reported symptoms in their hands and they generally expressed more negative perceptions than those without symptoms (three statistically significant instances). This pattern remained after stratification by age and exposure. When stratifying the material by company size, differences in the responses depending on whether the carpenters reported experiencing symptoms or not could mainly be seen in those employed at medium-sized companies.

**Table 2.** Average scores and Mann–Whitney comparisons between carpenters’ age, company size, estimated level of daily vibration exposure, presence of hand symptoms and total carpenter group

H&S management questions in the questionnaire	Age		Company size		Exposure		Hand symptoms		Total group (*n* = 194)
	Young^a^ (*n* = 99)	Old (*n* = 95)	Medium (*n* = 104)	Large (*n* = 90)	Lower^b^ (*n* = 98)	Higher (*n* = 94)	No (*n* = 79)	Yes (*n* = 115)	
Do you receive the information you need regarding vibration to be able to carry out your job in a safe manner?	2.6	3.1**	2.8	3.0	3.0	2.7	3.0	2.8	2.9
Do you experience that the work environment regulations and routines for preventing injuries function in reality?	2.7	3.2*	2.9	3.0	3.2	2.7**	3.1	2.8	2.9
If deficiencies in the work environment are detected, do you think improvements are then made?	3.3	3.7*	3.4	3.6	3.7	3.4*	3.7	3.4*	3.5
Do you think the management actively encourages safe work?	3.8	4.2**	3.9	4.2*	4.1	3.9*	4.1	3.9	4.0
Do you feel employees are encouraged to put forward ideas and suggestions for improvements concerning the work environment?	3.5	3.8*	3.5	3.7	3.7	3.5*	3.7	3.6	3.6
Do you experience that you can say what you think about how the work is performed, for example, concerning work associated with vibration?	3.7	4.0	3.8	3.8	4.0	3.6*	4.0	3.7*	3.8
Do you think the employer pays attention to, and takes seriously, the problems that arise on the job regarding the work environment and work safety?	3.6	3.9**	3.7	3.9*	3.9	3.6*	3.9	3.7	3.8
Do you experience that you generally talk about how the work can be improved in order to lead to a safer and healthier work environment?	3.5	3.8*	3.5	3.8**	3.7	3.6	3.8	3.6	3.7
Does your immediate foreman encourage orderliness on the job?	3.8	4.0	3.8	4.0	4.0	3.8	3.9	3.9	3.9
Do you think that your foreman considers safety to be part of daily work?	3.8	4.2***	3.9	4.2**	4.1	3.8*	4.0	4.0	4.0
Do you feel you have an influence on working conditions in your work?	3.9	4.1*	3.9	4.1*	4.1	3.8*	4.1	3.8**	4.0
In general, do you experience that there is a high risk of injury in your work?^c^	2.1	2.2	2.0	2.3*	2.1	2.2	2.1	2.2	2.1

^a^<40 years.

^b^<5 m/s^2^.

^c^For this question, a score of 1 corresponds to the most negative answer.

Group differences are significant at ***P-value < 0.001, **P-value < 0.01, *P-value < 0.05, two-tailed.

Analysis with the Mann–Whitney *U*-test was performed to investigate whether there were any differences in the responses from carpenters with symptoms working at a medium-sized company and the rest of the carpenters. The responses from the group with symptoms at the medium-sized companies were more negative in all instances than those from the rest of the carpenters (average difference in average score = −0.37, 10 of 12 statistical significances).

A large proportion of the carpenters responded negatively to the question, ‘Do you receive the information you need regarding vibration, to be able to carry out your job in a safe manner?’ Older carpenters were found to be more positive than younger ones, but no significant differences were found between the other subgroups. Another question eliciting many negative responses was, ‘Do you experience that the work environment regulations and routines for preventing injuries function in reality?’ Both younger carpenters and the more exposed carpenters responded more negatively to this question. Regarding the question ‘Do you feel you have an influence on the working conditions in your work?’ two subgroups stood out, showing more negative responses: carpenters with symptoms working at medium-sized companies and carpenters with symptoms and with high exposure. Three-quarters (74%) of the carpenters felt there was a high risk of injury at work. The average score for this H&S item was low for all analysed subgroups (Table 2). Regardless of the level of exposure or experience of symptoms, the majority of the carpenters reported experiencing a high risk of injury as a result of their work.

## Discussion

The carpenters were generally positive concerning the management’s commitment to H&S. This is important as the majority (74%) believed that there was a high risk of injury associated with their work. However, 40% of the carpenters reported that they did not receive adequate information on vibration exposure, and 36% felt that H&S regulations and routines were not adequately implemented in practice. Older carpenters, employees at larger companies and carpenters without hand symptoms or with lower vibration exposure had more positive perceptions of H&S management.

A strength of this study is that all 194 invited carpenters chose to participate. A limitation is that only two large and two medium-sized companies were included. Including more companies and smaller ones would have strengthened the study and may have shown greater differences in results. Only questionnaire data were used; interview results would have enriched the study.

It can be questioned whether the positive results reflect the real working situation at construction sites, as many employers do not comply with vibration legislation [7–9]. Studies in the Swedish construction industry reveal an existing strong focus on accident prevention, but considerably less on preventing long-term health risks [22]; thus, the respondents may have had accident prevention in mind when answering the questions.

Motivated management that prioritizes H&S in the workplace is important. The results suggest that management discuss the working environment and encourage carpenters to work safely. However, this positive attitude must be reflected in the practical implementation of measures. It has been reported that routines often fail in practice, even when H&S programmes are formally well organized [22]. The results indicated deficiencies as one-third of the carpenters felt H&S measures were not adequately implemented, and half of them reported a vibration exposure above the limit value. A lack of information and training on performing vibrating work tasks in a safe and healthy manner is likely to lead to less knowledge about vibration exposure and the risk of injury. The involvement of employees in H&S efforts is central in relation to safety [16]. Maloney *et al.* highlighted that whether a person becomes involved or not depends on their capability to be involved. Capability includes a person’s knowledge, abilities and skills, and can be developed through training [23]. A lack of knowledge can thus influence carpenters’ involvement in H&S. Poor knowledge concerning vibration exposure can also affect the worker’s capability to influence their exposure by making informed choices, for example, about what equipment to use. The carpenters in this study perceived they could influence their working conditions; however, those with symptoms working in medium-sized companies or were highly exposed were less positive. As construction sites are hazardous, it was not surprising that most carpenters perceived a high risk of injury. Interestingly though, they saw the risk of injury as high regardless of the magnitude of their vibration exposure or whether they had symptoms or not.

Younger carpenters were less positive concerning management’s safety efforts. Younger carpenters should have received information on H&S during their vocational training [24] and may therefore know more about employer’s obligations. Additionally, their negative perception may be due to younger and less experienced carpenters often performing more repetitive tasks that are ergonomically monotonous, leading to higher vibration exposure [25]. Older workers may have experienced much less focus on H&S, and therefore perceive current practices more positively. Carpenters with symptoms reported less influence over their working conditions than those without symptoms. The occurrence of symptoms in the hands may alert carpenters to the fact that their work involves a risk of vibration injury. Impaired hand function as a result of vibration may considerably impact work performance, preventing tasks from being performed in the usual manner. This could negatively affect the employee’s ability to work safely. H&S management may be more visible in larger companies as more resources are available, allowing formal systems to be developed and the appointment of H&S representatives. Nordlöf *et al.* [26] stated that underlying factors, such as structure, knowledge and available recourses, vary with the size of the company. This highlights the difficulties for smaller companies to maintain H&S efforts. The fact that carpenters with symptoms working in medium-sized companies gave more negative responses may indicate that they do not perceive the same management support as carpenters in larger companies. A large proportion of the carpenters reported a use of vibrating equipment such that their exposure was above the limit value. Those with higher exposure also responded more negatively to the H&S items. This could indicate that highly exposed carpenters perceive their work environment as more negative, or that carpenters who are dissatisfied with their work environment are more likely to overestimate their exposure. It has indeed been shown that self-reported duration of exposure is often overestimated [27,28]. Workplaces with high levels of vibration exposure could also have poorer H&S programmes. However, hand–arm vibration is always present in construction work, and can be difficult to avoid, despite otherwise good H&S measures.

Carpenters perceived a high risk of injury in their work but were generally positive about their work environment. This is despite the fact that some employers do not comply with legislation regarding education, risk assessments and medical surveillance [7–9]. At the same time, the risk of incurring a vibration injury is often overshadowed by more prominent risks on construction sites. This underlines the importance of raising awareness about vibration exposure, vibration injuries and the employer’s legal obligations. It also shows that there is a need to improve proactive H&S management at construction sites. Further research is needed on the practical implementation of vibration measures at work sites to overcome aspects that lead to harmful vibration exposure.
